# Central serous chorioretinopathy and heart rate variability analysis with a smartphone application

**DOI:** 10.1038/s41598-020-71938-3

**Published:** 2020-09-11

**Authors:** Keigo Takeshima, Koji Tanaka, Ryusaburo Mori, Yu Wakatsuki, Hajime Onoe, Takuya Sakakibara, Yorihisa Kitagawa, Hiroyuki Nakashizuka, Norihiro Tsuchiya

**Affiliations:** 1 Division of Ophthalmology, Nihon University School of Medicine, 1-6 Kandasurugadai Chiyoda-ku, Tokyo, Japan; 2 Department of Ophthalmology, Diabetes Center, Tokyo Women’s Medical University, Tokyo, Japan; 3 Omotesando Internal Medicine and Ophthalmology Clinic, Internal Medicine, Tokyo, Japan

**Keywords:** Retinal diseases, Risk factors

## Abstract

The purpose of this study was to quantitatively analyze heart rate variability (HRV) in patients with central serous chorioretinopathy (CSC) by using a smartphone-based application (ANBAI: DUMSCO Inc.) for measurement, and to clarify its relationships with CSC. The subjects were 64 CSC patients (mean age 48.7 ± 7.6 years, 57 males and 7 females). After providing consent, the patients downloaded ANBAI apps to their smartphones. HRV was measured by photoelectric volume pulse wave measurement with a smartphone camera each morning for a minimum of 1 week. The primary outcome was to analyze HRV by calculating log LF/HF (Low Frequency/High Frequency components), an index of autonomic tone, which was then compared with a control group of 35,226 individuals from the application. Secondary outcome measures included disease duration, body mass index, exercise habits, smoking history, steroid use, occupation, lifestyle regularity, psychological fatigue, physical fatigue, and average sleep time. The log LF/HF was significantly higher in the patient group than in the control group (*P* < 0.001). Log LF/HF was significantly lower in patients with exercise habits as a factor contributing to log LF/HF in the patient group (*P* = 0.019). Analysis of HRV in CSC patients showed an impairment of the autonomic nervous system. Exercise habits may also be associated with CSC.

## Introduction

Central serous chorioretinopathy (CSC) is a disorder characterized by serous retinal detachment in the macular region, mainly affecting men in their 30s-50s. Stress, smoking, type A behavior, and a history of steroid use have been reported as risk factors^1–9^.

Symptoms include altered vision and decreased vision, but spontaneous resolution is often observed within 3 months. However, when CSC recurs and is prolonged, it causes irreversible loss of visual acuity and therefore requires treatment using retinal photocoagulation and photodynamic therapy. Other reports have included the use of subthreshold lasers and the use of Eplerenone^10–12^.

Levels of endogenous adrenocortical hormone (cortisol) have been reported as an indicator of stress^13^. Type A behavior was reportedly common in interviewed subjects, and CSC develops when laboratory animals are subjected to stress^14^.

**Figure 1:**
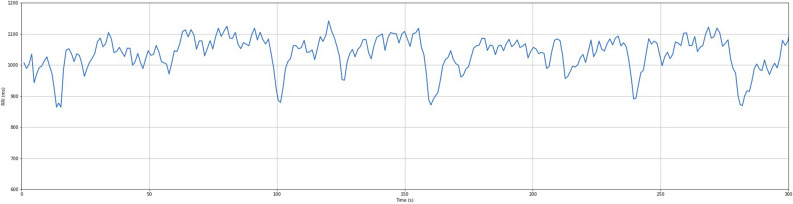
Heart rate variability waveform. This figure shows data for heart rate variability time recorded over a 300 s period under resting conditions in a healthy individual. Units on the horizontal axis are times (s) and units on the vertical axis are heart rate intervals (ms).

**Figure 2:**
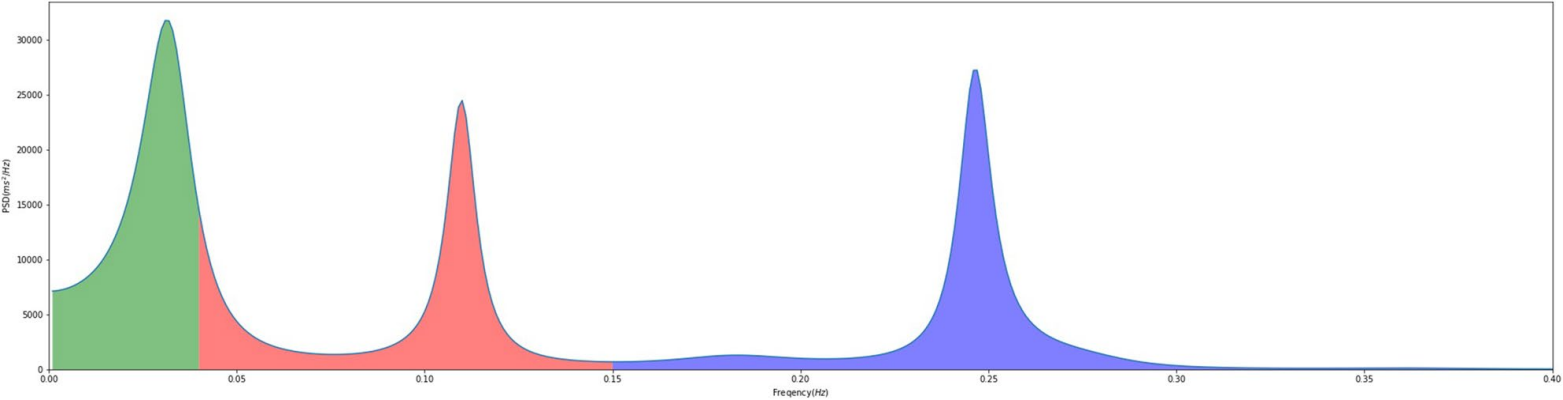
Power spectral density and spectral components. LF (Low Frequency) is represented by the red area, and HF (High Frequency) by the blue area. To measure the areas of the HF and LF components, the LF component region of the power spectrum (0.05 Hz to 0.15 Hz) and the intensity of the HF component region (0.15 Hz to 0.40 Hz) are combined.

The heart rate interval of the electrocardiogram is not always constant but is variable, and the periodic variation of the heart rate interval is termed the heart rate variability (HRV). HRV allows the autonomic nervous system tone of the heart to be determined noninvasively by measuring the variability of each heartbeat. Sympathetic and parasympathetic nerve activities are reflected in the heart rate, and fluctuations in HRV reflect the balance between the two. Decreased HRV indicates increased sympathetic and decreased parasympathetic tone. Decreased HRV has been reported in aging^15^, diabetic neuropathy^16–18^, heart failure^19^, and acute myocardial infarction^20,21^. There are various factors contributing to HRV, a time domain index and a frequency domain index are both components of the analysis method. The frequency domain indicator represents the magnitude of the variation as a function of frequency, with the low frequency region (0.04 to 0.15 Hz) designated LF (Low Frequency) * and the high frequency region (0.15 to 0.4 Hz) designated HF (High Frequency) *. The LF/HF* ratio is an index for sympathetic function assessment (Figs. 1, 2). The LF component reflects the baroreceptor system and is affected by sympathetic and parasympathetic nerves, while the HF component reflects respiratory fluctuations and is affected by parasympathetic nerves modulating respiration. The relaxed state is predominantly parasympathetic, with the emergence of both HF reflecting respiratory variability and LF reflecting blood pressure variability.

The HF which reflects fluctuations in respiration cannot be transmitted under stress conditions, however, in which the sympathetic nervous system shows dominance. As HF decreases, the LF component, which reflects blood pressure fluctuation, persists. Thus, the LF/HF ratio increases because HF decreases in the stressed state, while HF increases in the relaxed state at the same LF/HF ratios, such that the LF/HF ratio ultimately decreases. Utilizing this phenomenon, Bernasconi et al. reported an association of stress with CSC using LF/HF, a frequency-domain index obtained by measuring HRV using a Cardiometer^22^.

The equipment which we used for measurement of HRV is extremely convenient and allows relationships with various diseases to be demonstrated.

With the advent of smartphone photoplethysmography (smartphone PPG) employing smartphones, currently in use worldwide, HRV can now be measured easily. This measurement method allows most of the indices which can be calculated from the conventional measurement methods to be determined, and the requirement for only a commercial smartphone greatly simplifies the process^23,24^.

This study aimed to measure HRV by using a smartphone application (ANBAI: DUMSCO Inc.) to quantify autonomic function in patients with CSC, and thereby clarify the relationship of HRV with CSC.

*Additional description.

- LF (low frequency; ms^2^)

The power spectrum of the 0.004 ~ 0.15 Hz frequency band.

This value reflects both (vasomotor) sympathetic and parasympathetic activity.

The parasympathetic influence on this frequency band appears in the LF while breathing is deep – less than or equal to 9 breaths per minute (0.15 Hz).

Thus, LF levels are very high during slow, regular breathing in a relaxed state, implying an increase in parasympathetic, rather than an increase in sympathetic, activity.

- HF (high frequency; ms^2^)

The power spectrum of the 0.15 ~ 0.4 Hz frequency band.

This level reflects the activity of the parasympathetic (vagus) nerve.

HF is known as RSA (respiratory sinus arrhythmia), and is also known as the respiratory zone because it shows variations in the RR interval due to breathing.

Heart rate increases with inspiration and decreases with exhalation.

Slow regular breathing increases the amplitude of the HF peak in the power spectrum.

- LF/HF ratios

The ratio of the power of LF (low frequency) to HF (high frequency).

This value represents the overall balance between sympathetic and parasympathetic nerves. Higher values indicate a sympathetic predominance, whereas lower values indicate a parasympathetic predominance. However, it should be noted that it reflects an increase in parasympathetic activity due to the high RSA (breathing) effect during regular deep breathing.

## Methods

HRV analysis using smartphone PPG:

Assessments were made using a smartphone-based photoelectric volumetric pulse wave assay (smartphone PPG).

**Figure 3:**
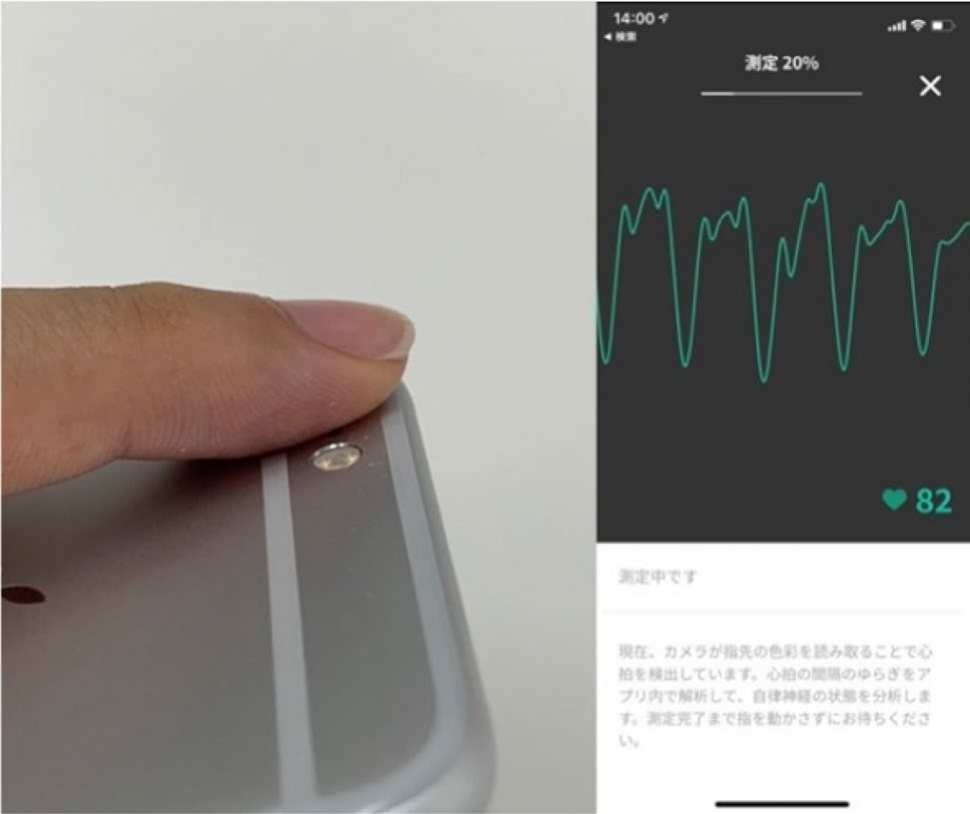
How to measure heart rate variability with a smartphone. The smartphone application (ANBAI: DUMSCO, Inc.) uses the camera (CMOS; complementary metal-oxide semiconductor) attached to the smartphone as a light receptor and the LED (Light Emitting Diode) flashlight as a light source to make the smartphone an optical electric volume pulse wave measuring device, and the data thus obtained can be used to perform heart rate variability analysis.

This smartphone application (ANBAI: DUMSCO, Inc.) is a device that uses a camera (CMOS (complementary metal-oxide semiconductor)) mounted on a smartphone body as a light receptor and a LED (Light Emitting Diode) flashlight as a light source making the smartphone an optoelectrical volume pulse wave measuring device. The data obtained can be used to perform HRV analysis.

(Fig. 3) Characteristically, the single-measurement time is as short as approximately 1 min, indicating a noninvasive and simple process.

Specifically, a finger is applied to the camera site on a smartphone to measure the heartbeat.

HRV analysis, known as biophenomena^25^ reflecting autonomic activity, is the fluctuation of RR intervals (RR interval: RRI) by electrocardiography.

HRV is an index of autonomic tone in the heart, and can be measured directly. The analytical methods for HRV analysis include time-domain indices, which represent the magnitude and the magnitude of variation as a function of time or heart rate. The LF/HF ratios are computed as described below.

Power spectral densities are computed to extract periodic structures from HRV data. The power spectral density represents the periodic waves constituting the time series data, together with their constituent ratios.

To estimate autonomic balance from HRV, we extract the HF component corresponding to respiratory variability and the LF component corresponding to the Mayer wave, indicating blood pressure variability, from time series data of HRV and then compare the magnitudes of these two parameters.

To measure the magnitudes of the HF and LF components, the LF component region of the power spectrum (0.05 Hz to 0.15 Hz) and the intensity of the HF component region (0.15 Hz to 0.40 Hz) are summed (yielding an integrated value).

The log LF/HF value is used herein as an index of sympathetic function.

### Study design

Inclusion criteria: Among consecutive CSC patients who attended the Nihon University Hospital from June to October 2019, we invited all who had a smartphone to participate in the study. Participating patients had serous retinal detachments (SRD) at their first visit, and only those who agreed to the study and were free of choroidal neovascularization (CNV) by optical coherence tomography (OCT), fluorescein angiography (FA), and indocyanine green angiography (ICGA), OCT angiography, and who could deny other diseases, were included. We also defined acute CSC as onset within 3 months, and chronic CSC as onset more than 3 months previously. We asked the potential subject to download the app to his or her smartphone. Among them, 64 patients (mean age 48.7 ± 7.6 years, 57 males and 7 females) in whom HRV was measured in the morning, upon awakening, and who met the criterion for measurement accuracy of 90% or more were included in the study.

Only data with a measurement accuracy of ≧ 90% were extracted, and always just as patients were awakening in the morning and were in the recumbent position before eating. The application was used at least once a day for a minimum of 1 week, consistently in the morning.

The control group was based on data from 35,226 individuals (23,241 men, 11,985 women; mean age 41.4 years) accumulated by ANBAI apps in the morning, with a measurement accuracy of 90% or greater.

Log LF/HF was compared between the patient and control groups (primary endpoint).

### Secondary endpoints (interview)

Exercise habits in the patient group (question: do you exercise more than 30 min twice a week?—recorded as 1: yes, 2: no), current smoking (1: yes, 2: no), inhalants and oral steroid use (1: yes, 2: no), work content (1: clerical, 2: outdoors ; construction site, farming, etc.), 3: driver (taxi, bus, train), 4: other, well-regulated life (1: yes, 2: no), psychological exhaustion (1: no, 2: minimal, 3: yes), physical exhaustion (1: no, 2: minimal, 3: yes), and mean sleeping times were collected. Patients were also interviewed about their exercise habits, smoking history, steroid use, occupation, the regularity of their lifestyles, psychological fatigue, physical fatigue, and average sleep duration.

Exercise habit was set as the standard for the exercise habit parameter, since exercising for more than 30 min per day for more than 2 days per week is recommended (4METS; Metabolic Equivalents・hours/week) for treating and ameliorating diseases such as hypertension and diabetes mellitus^26,27^.

### Ethics statement

This study adhered to the tenets of the Declaration of Helsinki. This was a retrospective, single center study, and the procedures used were approved by the Ethics Committee of the Nihon University Hospital, Tokyo, Japan (Approval 5/Dec/2019).

### Informed consent

Informed consent was obtained from all individual patients included in this study.

## Results

Ninety-one consecutive CSC patients who visited the University of Japan Hospital during the period from June to October 2019 and who provided consent for the examination were considered for enrollment in this study. All 91 CSC patients showed SRD. Only patients with no CNV on OCT, FA, and ICGA, OCT angiography, and who could deny other diseases were included. Among them, 64 patients (mean age 48.7 ± 7.6 years, 57 males and 7 females) in whom HRV was measured in the morning, upon waking, and who met the criterion for measurement accuracy of 90% or more were included in the study.

The mean values of age, refraction error, disease duration, Body Mass Index (BMI), foveal retinal thickness (central retinal thickness: CRT), foveal choroidal thickness (central choroidal thickness: CCT), and ETDRS (Early Treatment Diabetic Retinopathy Study) visual acuity at first visit are listed in Table 1. Because all patients had SRD, the CRT were increased, and the CCT were markedly increased, averaging 423 μm, due to the Pachychoroid of the diseases. There were no cases with an intraretinal fluid, but 27 cases out of 64 had PED (42%).

**Table 1:** Characteristics of patients. Values are presented as the mean ± standard deviation.

Age (year)	48.7 ± 7.6
Refraction (Diopter)	− 1.35 ± 3.5
Disease duration (months)	20.0 ± 24.6
BMI	23.47 ± 2.72
CRT (μm)	357.4 ± 135.1
CCT (μm)	423.8 ± 112.5
ETDRS visual acuity (letters)	81.1 ± 10.4

*BMI* Body mass index, *CRT* central retinal thickness, *CCT* central choroidal thickness, *ETDRS* Early Treatment of Diabetic Retinopathy Study.

The control group for the ANBAI app was comprised of 35,226 individuals (23,241 men and 11,985 women; mean age 41.4 years). The only data for the controls were sex and age. Because the Control Group was informed only by age and sex, they may have a systemic disorder. However, because the Control group has a large number of individuals (35,226) and the mean age is relatively young (41 years), any effect from other systemic diseases was considered negligible, if any. None of the patients had cardiac diseases such as atrial fibrillation or were taking β-blockers.

### Comparison between patient and control groups (primary endpoint)

Table 2 shows the results of comparisons of HRV parameters between the patient and control groups. The log LF/HF value was significantly higher in the patient than in the control group (*P* < 0.001). The value did not change even if adjusted the male/female ratio.

**Table 2:** Comparison of log LF/HF between patients and controls.

	Control	Patients	*P* value
	EMM ± SD	EMM ± SD	
log LF/HF	0.22 ± 0.01	0.34 ± 0.03	< 0.0001*

*EMM* estimated marginal mean, *LF/HF* low frequency/high frequency, *SD* standard deviation.

*P value < 0.05.

Examinations of the relationships between these items and log LF/HF revealed significant differences in exercise habits (Table 3). The log LF/HF values were significantly lower in patients who habitually exercised for ≧ 30 min twice a week (*P* = 0.019). CCT was not associated with log LF/HF. We then compared the log LF/HF values for acute and chronic CSC groups, and found there was no significant difference (Table 4).

**Table 3:** Associations between log LF/HF and the background factors of CSC patients.

	Mean	Relation to log LF/HF	
		ρ	*P* value
Age (years)	48.7 ± 7.6	− 0.054	0.671
Sex (male:female)	57:7	− 0.215	0.087
Disease duration (months)	20.0 ± 24.6	0.017	0.895
BMI	23.47 ± 2.72	0.067	0.599
Exercise habits^a^	45%	0.293	0.019*
Smoking (current)	27%	0.156	0.218
Steroids (in use)	19%	0.067	0.598
Job (%)			
Clerical	58%	0.302	0.793
Outdoors	28%	0.229	0.307
Driver	11%	0.544	0.059
Other	3%	0.359	0.970
Well-regulated life	36%	− 0.043	0.735
Psychological fatigue	2.07 ± 0.60	0.163	0.199
Physical fatigue	2.16 ± 0.61	0.200	0.112
Sleeping time (/day)	6.17 ± 0.59	− 0.008	0.948
CRT (μm)	357.4 ± 135.1	− 0.200	0.113
CCT (μm)	423.8 ± 112.5	− 0.180	0.155
ETDRS visual acuity (letters)	81.1 ± 10.4	− 0.087	0.497

^a^Exercise habits in the patient group (do you exercise more than 30 min twice a week?

(1: yes, 2: no), current smoking (1: yes, 2: no), inhalant and oral steroid use (1: yes, 2: no), Job (1: clerical, 2: outdoors (construction site, farming, etc.), 3: driver (taxi, bus, train driver), 4: other), well-regulated life (1: yes, 2: no), psychological fatigue (1: no, 2: minimal, 3: yes,) physical fatigue (1: no, 2: minimal, 3: yes), and mean sleeping times were collected by interview.

*BMI* Body mass index, *CSC* central serous chorioretinopathy, *CRT* central retinal thickness, *CCT* central choroidal thickness, *ETDRS* Early Treatment of Diabetic Retinopathy Study, *log LF/HF* Low Frequency/High Frequency, *ρ* Spearman's rank correlation coefficient.

*P value < 0.05.

**Table 4:** Comparison of log LF/HF between acute and chronic CSC.

	Acute CSC (n = 24)	Chronic CSC (n = 40)	*P* value
	Mean ± SD	Mean ± SD	
log LF/HF	0.48 ± 0.78	0.54 ± 0.59	0.728

*Acute CSC* within 3 months of onset, *Chronic CSC* more than 3 months after onset, *CSC* central serous chorioretinopathy; *LF/HF* low frequency/high frequency, *SD* standard deviation.

*P value < 0.05.

## Discussion

The evaluation was carried out employing the log LF/HF ratio for the purpose of the adjusting the distribution by logarithmic division of LF/HF^28^. Our results also showed that the group of patients with CSC had elevated log LF/HF as compared with the control group, suggesting autonomic dysfunction to play a role in CSC. Patients with CSC have autonomic dysfunction (stress), which is known to be a risk factor for CSC development and increased log LF/HF might be attributable to certain forms of stress.

Bernasconi et al. used LF/HF to divide patients with CSCs into four groups, i.e. acute, acute-relapse, chronic, and complete-remission, and compared them with the control group^22^. LF/HF levels were significantly elevated in the acute, acute relapse, and complete remission groups as compared with the normal control group. The pathogenesis of CSC is reportedly associated with increased sympathetic activity of autonomic nerves. In the current study, exercise habits, smoking habits, steroid use and occupation, well-regulated life, psychological fatigue, physical fatigue, and average sleep time data were collected from the patient group as secondary endpoints. The results showed that patients who habitually exercise for more than 30 min twice a week had significantly lower log LF/HF values. Therefore, it was suggested that patients with regular exercise habits had fewer autonomic function abnormalities. Autonomic function abnormalities manifest as an imbalance between sympathetic and parasympathetic nervous system activities, but patients with symptoms tend to have less parasympathetic than sympathetic activity^29^. Exercise habits are associated with a significantly higher parasympathetically regulated baroreceptor sensitivity^30^, which makes reducing parasympathetic activity difficult and thus unlikely to cause autonomic abnormalities. The ρ was 0.293 (*P* = 0.019), so that the correlation was weak. We hope to tackle this issue in the future by increasing the number of cases. We also investigated whether there were differences in the values of log LF/HF between acute and chronic CSC, but no significant differences were observed. However, given the modest number of subjects, in future studies, it will be necessary to examine an increased number of cases.

Choroidal vessels are innervated by three nerve types: sympathetic, parasympathetic, and sensory. Combined sympathetic and parasympathetic disorders have been reported to alter choroidal blood flow^31,32^. It has also been reported that there are changes in choroidal blood flow in CSC^33,34^. Therefore, choroidal blood flow change appears to be caused by the autonomic dysfunction affecting CSC patients.

In the present study, log LF/HF, a measurement of sympathetic function, was significantly elevated in patients with CSC. This may reflect increased sympathetic function, that is, stress conditions. We conveniently performed HRV analysis using a smartphone, rather than a conventional PPG. Smartphone-based PPG has both advantages and disadvantages. As an advantage, it is a measurement method that can be performed in daily life and rapidly employing a smartphone application. Disadvantages include the use of smartphones being limited to younger age groups and the challenges of having all patients manage the app daily.

Autonomic activity does not maintain constant tension throughout the day, instead showing a diurnal variation^35–37^. The LF component in the frequency domain is affected by various external factors, including physical and mental activities during the day, as well as body posture. Also, there are periodic and non-periodic variations in HRV^38^, as well as slow periodic variations below 0.05 Hz at night^39^. Nocturnal HRV is characterized by increased parasympathetically mediated respiratory fluctuations at HF, while increased sympathetic activity concomitant with wakefulness and diminished nocturnal hyper parasympathetic activity is seen during the night. Therefore, it is important to employ a single measurement time point, such as in the morning upon awakening, when heightened sympathetic nerve activity occurs.

As mentioned above, HRV is affected by a variety of activities including diet, body position, breathing, stress, and sleep. After a meal, stimulation by insulin secretion increases sympathetic nerve activity and increases blood flow to the viscera, thereby decreasing venous return and peripheral circulatory resistance, leading to a decrease in parasympathetic nervous system activity^40,41^. This can be demonstrated by the head-up tilt test showing autonomic nerve activity to be highly affected by the position of the body, and that there is a shift to sympathetic nervous system dominance in the standing position, while parasympathetic nervous system dominance is seen in the decubitus position. This test shortens the average RR interval and decreases the amplitude of the HF component^42,43^.

An inhibitory effect is exerted on parasympathetic nerve activity due to reduced venous return.

Breathing rate and tidal volume significantly affect HRV, and the HF component decreases because tidal volume decreases due to an increase in the respiratory rate^44^. Psychiatric stress increases sympathetic tone, markedly increases LF components, and decreases in HF components^45,46^. Heart rate decreases during sleep due to decreased sympathetic activity and increased parasympathetic activity. Thus, the amplitudes of the HF and LF components increase and LF/HF ratios decrease^47^. Considering these factors involved in HRV, we attempted to extract the effects of stress by minimizing confounding factors by obtaining measurements with the subjects in the supine position after they had awakened and before they had eaten.

Taking these considerations together, we performed HRV analysis of CSC patients using a convenient smartphone-formulated PPG. Compared with the large number of controls, log LF/HF, a sympathetic index, was significantly elevated in the CSC patients.

Since measurement is convenient and the app can be downloaded, use by large numbers of people is feasible, allowing us to potentially reduce the risk of CSC.

Limitations of this study include the small number of CSC patients who underwent measurement, the absence of physician observation at the time of measurements, the availability of only gender and age information in the control group, and the lack of smartphone PPG quality control.

These promising preliminary findings merit further research.

### Statistical analysis

This study collected data from the same subject multiple times. The HRV parameters (mainly, the LF/HF ratio) were analyzed with linear mixed models that included group (control vs. patients) as a fixed effect, and the subjects as a random effect. The results were reported as the estimated marginal mean (EMM) and standard deviation (SD) for each group.

The association between log LF/HF values and respective factors (Table 3) used the Spearman correlation. When these factors were corrected by multiple regression analysis, they were all considered to be non-significant in the test due to the small number of samples. This analysis was not adjusted because it was positioned as an exploratory analysis**.** This is a hypothesis-generating type of study and further confirmatory studies are needed to prove their hypothesis. A *P* value of < 0.05 was considered to indicate a statistically significant difference, and all *P* values were two-sided. All statistical analyses were performed using SPSS version 23.0 (IBM Japan, Ltd., Tokyo, Japan).

## Data Availability

The datasets of this study are available from the corresponding author upon reasonable request.
